# Mr. White, on Hydrocephalus

**Published:** 1800-04

**Authors:** W. White

**Affiliations:** Bath


### *To the Editors of the Medical and Phyſical Journal.*

GENTLEMEN,

I Have taken the liberty of communicating to you the following caſe of Hydrocephalus Internus, (it having occurred ſince I ſent the other caſes) if you think it worth inſerting in the Medical and Phyſical Journal. I remain,

GENTLEMEN,

Your obliged and humble Servant,

*Bath, Feb.* 8, 1800. W. WHITE.

---

ANNE SPENCER, aged 39, of a common ſanguine temperament, who had had occaſional attacks of head-ach upwards of four months, attended with chillineſs and epileptic fits; but did not come under my immediate care till within nine days of her death: ſhe then complained of a violent head-ach, and could not bear an erect poſture; the fits alſo recurred very often, although they only continued a few minutes. There was no unnatural appearance of the eyes, except that I obſerved, two days prior to her death, the pupil of the left eye to be more dilated than that of the right; the next day, however, it appeared the ſame as the other: and it may be worthy of remark, that ſhe even appeared to retain her ſight till within a few minutes of her death. Except during the fits, ſhe appeared ſenſible. Her appetite was very good, but ſhe ſometimes vomited; her pulſe, during the whole of the time, was preternaturally ſlow; tongue was clean; bowels were coſtive. The application of leeches, bliſters, &c. appeared to relieve her much from the violence of the pain, but the diſeaſe terminated fatally in a fit of apoplexy.

On examining the head, the veſſels of the *dura* and *pia ma-*

ter were found extremely turgid with blood; and about two ounces of clear fluid were found in the ventricles, which was neither coagulable by heat, nor by the concentrated acids.

---

